# 646. Subgroup Analyses of Microbiological Cure Rates by Baseline Zoliflodacin MIC and Susceptibility to Ciprofloxacin in Participants from the Global Zoliflodacin Phase 3 Randomized Controlled Trial

**DOI:** 10.1093/ofid/ofaf695.210

**Published:** 2026-01-11

**Authors:** Sarah McLeod, Esther Bettiol, Varalakshmi Elango, Khurram Rana, Drew Lewis, Alison Luckey

**Affiliations:** Innoviva Specialty Therapeutics, Inc., Waltham, MA; Global Antibiotic R&D Partnership, Geneva, Geneve, Switzerland; Global Antibiotic R&D Partnership, Geneva, Geneve, Switzerland; Innoviva Specialty Therapeutics, Madison, Connecticut; Innoviva Specialty Therapeutics, Madison, Connecticut; Global Antibiotic R&D Partnership (GARDP), Geneva, Geneve, Switzerland

## Abstract

**Background:**

Zoliflodacin (ZFD) is a first-in-class spiropyrimidinetrione gyrase inhibitor with a novel mode of action and *in vitro* activity against multidrug-resistant *Neisseria gonorrhoeae*. In a global randomized controlled Phase 3 trial, a single oral dose of ZFD demonstrated noninferiority compared to dual therapy of ceftriaxone and azithromycin for treatment of uncomplicated urogenital gonorrhea in the primary (micro-ITT) analysis set. Cure rates for extragenital infections were comparable between treatment arms. Here, subgroup analyses of microbiological cure rates by baseline ZFD MIC and susceptibility to ciprofloxacin (CIP) are presented.

**Figure:**
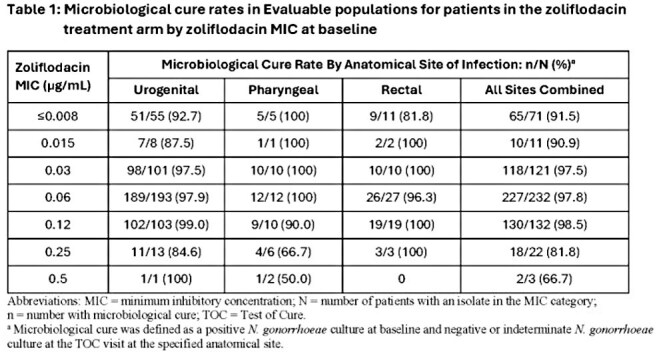

**Figure:**
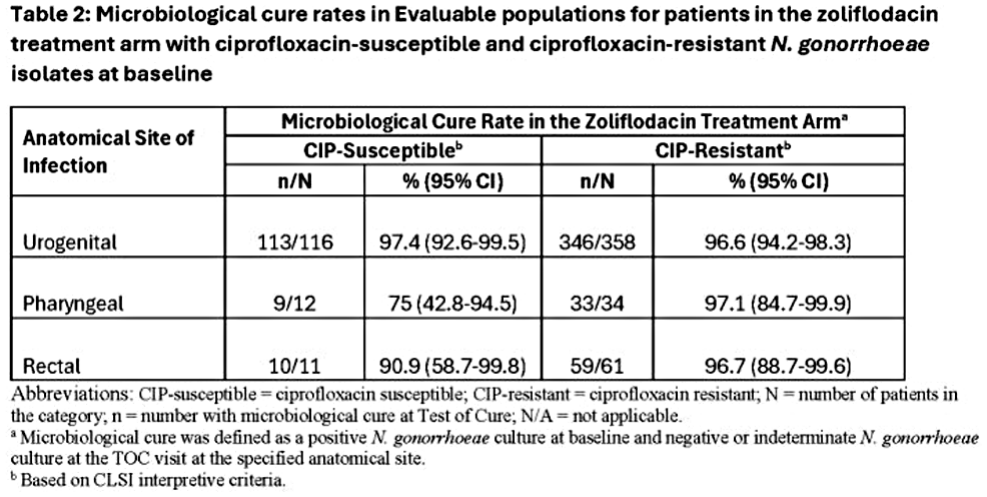

**Methods:**

Microbiological cure was determined by culture from urogenital, rectal and pharyngeal sites at Test of Cure (TOC; Day 6±2) in the Evaluable population (patients with a positive baseline *N. gonorrhoeae* culture and a culture result at TOC). Susceptibility of baseline *N. gonorrhoeae* isolates was determined by agar dilution following CLSI guidelines. Data were analyzed by anatomical site, treatment arm, MIC of ZFD, CIP susceptibility, geographical location, and sex assigned at birth.

**Results:**

Baseline *N. gonorrhoeae* isolates from participants treated with ZFD had ZFD MICs of ≤ 0.008-0.5 µg/mL. For urogenital, rectal and pharyngeal sites combined, microbiological cure rates were high at ZFD MICs ≤ 0.25 µg/mL (Table 1). There were too few isolates with a MIC of 0.5 µg/mL (n=3) to infer a relationship between this MIC and cure rate. Microbiological cure rates at urogenital sites of infection for participants treated with ZFD were 96.6% (346/358; CI: 94.2-98.3) for patients with CIP-resistant and 97.4% (113/116; CI: 92.6-99.5) for CIP-susceptible baseline *N. gonorrhoeae* (Table 2). High rates of microbiological cure for CIP-resistant *N. gonorrhoeae* were maintained for pharyngeal and rectal sites of infection, geographical location and sex at birth.

**Conclusion:**

High rates of microbiological cure were observed in participants who received a single, oral dose of ZFD and had baseline isolates with ZFD MICs ≤ 0.25 µg/mL or that were CIP-resistant, regardless of anatomical site of infection, geographical location and sex assigned at birth. These results support the continued development of ZFD for the treatment of uncomplicated gonorrhea.

**Disclosures:**

All Authors: No reported disclosures

